# Transmission dynamics and successful control measures of SARS-CoV-2 in the mega-size city of Guangzhou, China

**DOI:** 10.1097/MD.0000000000027846

**Published:** 2021-12-03

**Authors:** Hongjun Zhao, Zhoubin Zhang, Wenhui Lun, Zongqiu Chen, Xiaoxiao Lu, Jingrong Li, Fuman Qiu, Shunming Li, Chun Mao, Ying Lu, Jinbin Chen, Qing He, Jiachun Lu, Zhicong Yang

**Affiliations:** a State Key Lab of Respiratory Disease, The First Affiliated Hospital, Institute for Public Health, Guangzhou Medical University, 195 Dongfengxi Road, Guangzhou, PR China.; b Guangzhou Centre for Disease Control and Prevention, Guangzhou, PR China.; c Department of English and American Studies, Faculty of Languages and Literatures, Ludwig Maximilian University (LMU), Munich, Germany.

**Keywords:** control measures, novel coronavirus disease, severe acute respiratory syndrome coronavirus 2, the effectiveness of public health interventions, transmission dynamics

## Abstract

The severe acute respiratory syndrome coronavirus 2 has caused a worldwide pandemic. Control measures differ among countries and have a varying degree of effectiveness, which requires assessment. To evaluate the effectiveness of public health interventions of the coronavirus disease 2019 (COVID-19) in Guangzhou by 3 periods according to interventions: January 7 to 22 (no intervention), January 23 to February 23 (implemented intensive interventions), and February 24 to May 17 (the normalization mode of COVID-19 prevention and control).

We collected the information of 745 COVID-19 patients and their close contacts as well as control measures in Guangzhou from January 7 to May 17, 2020. We estimated the epidemiological characteristics, disease spectrum of COVID-19 cases, key time-to-event intervals, and effective reproduction number over the 3 periods. The basic reproduction number of severe acute respiratory syndrome coronavirus 2 was also calculated over period 1.

Approximately 45.8%, 49.8%, and 4.4% of cases from close contacts were asymptomatic, symptomatic, and severe, respectively. The median incubation period was 5.3 days (the percentiles of 2.5–97.5, 1.5–18.4 days) and the median serial interval fitted with gamma distribution was 5.1 days (the percentiles of 2.5–97.5, 0.8–15.9 days). The estimated median of onset-to-quarantined time in Period 1 to 3 were 7.5, 3.4, and 2.9 days (the percentiles of 2.5–97.5, 2.1–14.2, 3.9–14.7, and 6.0–20.0 days) respectively and the median of onset-to-confirmation time in period 1 to 3 were 8.9, 4.9 and 2.4 days (the percentiles of 2.5–97.5, 2.6–16.6, 0.9–14.6, and 0.5–11.8 days). In period 1, the reproduction number was 0.9 (95% confidence interval, 0.5–1.4) and fluctuated below 1.0 before January 22 except for January 14. The effective reproduction number gradually decreased in the period 2 with the lowest point of 0.1 on February 20, then increased again since March 27 and reach a spike of 1.8 on April 12. The number decreased to below 1.0 after April 17 and decreased further to <0.2 after May 7 in the period 3.

Under prospective dynamic observation, close contacts turned into infected cases could provide a spectrum of COVID-19 cases from real-world settings. The lockdown of Wuhan and closed-loop management of people arriving Guangzhou were effective in halting the spread of the COVID-19 cases to Guangzhou. The spread of COVID-19 was successfully controlled in Guangzhou by social distancing, wearing a face mask, handwashing, disinfection in key places, mass testing, extensive contact tracing, and strict quarantine of close contacts.

## 1 Introduction

The ongoing pandemic of the novel coronavirus disease (COIVD-19), caused by the novel severe acute respiratory syndrome coronavirus 2 (SARS-CoV-2) purportedly from bats, has now affected 220 countries and areas worldwide since the end of 2019.^[1,2]^ As of November 20, 2020, >56 million confirmed cases and over 1 million deaths had been reported.^[2]^ In China, the COVID-19 epidemic was under control after Chinese authorities taking a series of unprecedented measures to control the transmission, including screening of high-risk populations, prompt identification and reporting of suspicious cases, and rapid diagnosis of cases, which effectively suppressed the spread of SARS-CoV-2.^[3,4]^ But the COVID-19 cases and deaths worldwide still are rising. As cold weather moves in, where the clusters outbreak is not under control, the COVID-19 pandemic will continue.^[5]^

**Figure 1:**
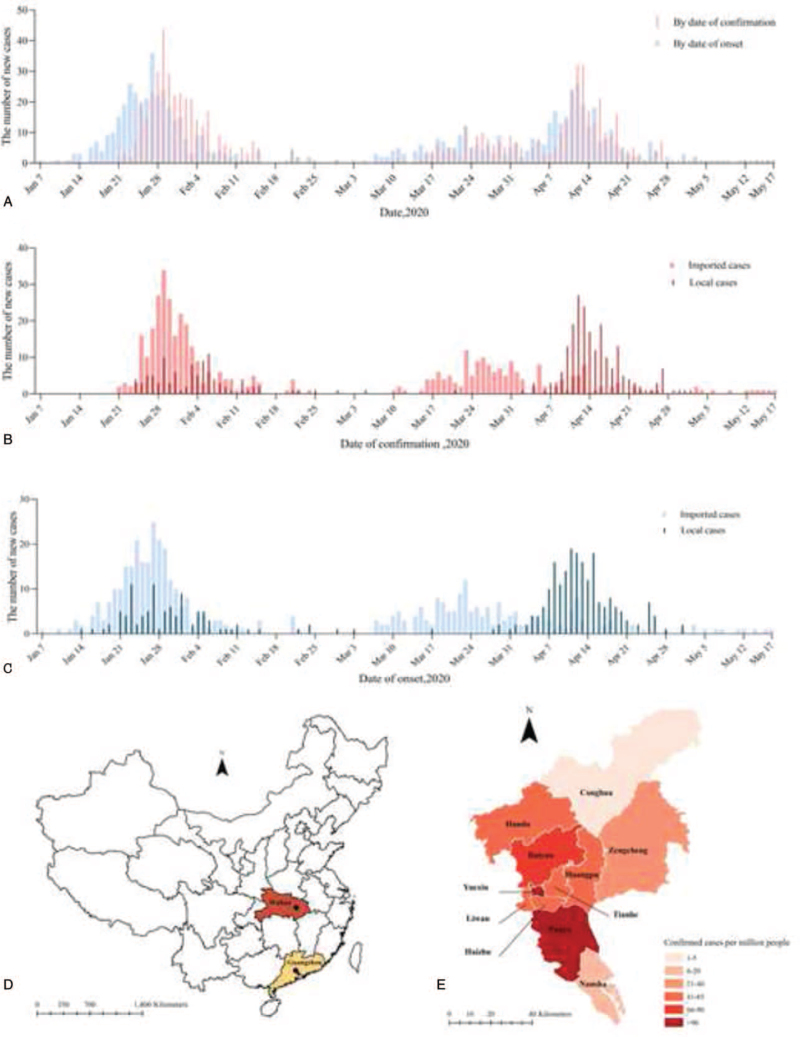
Temporal and spatial distribution of COVID-19 cases in Guangzhou, China, 2020. (A) The time distribution of COVID-19 cases by date of confirmation and onset; (B) the time distribution of imported and local cases by date of confirmation; (C) the time distribution of imported and local cases by date of onset; (D) location of Guangzhou and Wuhan, China; (E) spatial distribution of COVID-19 cases rate per million people across Guangzhou, China.

Guangzhou, the capital city of Guangdong Province, is located in the south of China (see Fig. 1D, which illustrates the location of Guangzhou and Wuhan, China. Circles indicate capital cities). It is one of the national central cities in China, with a developed economy and convenient transportation. The city covers an area of 7434.4 km^2^, consisting of 11 administrative districts, with a registered population of 9.5 million as well as migrants of 5.8 million and a foreigner population of 84,000,^[6]^ which might lead to a large number of imported cases in Guangzhou. The first imported cases showed symptoms on January 21 and the first overseas imported case was confirmed on March 11, which lead to the second COVID-19 outbreak in Guangzhou. In our study, we analyzed the spatial, temporal, and population distribution of all 745 COVID-19 cases in Guangzhou as of May 17, 2020 to describe the epidemiological characteristics and transmission dynamics of COVID-19 and evaluated the effectiveness of prevention and control measures for providing control policy recommendations for countries and areas to combat the global pandemic of COVID-19.

## 2 Method

### 2.1 Data sources and collection

A total of 745 laboratory-confirmed COVID-19 reported between January 21, 2020 and May 17, 2020 were included in our study and were gathered by Guangzhou Centre for Disease Control and Prevention (CDC). The epidemiological investigations of cases and their close contacts were conducted by county-level CDCs using a standardized investigation. We extracted the demographic characteristics (sex, age, regions), clinical data (symptoms and disease severity), epidemiological characteristics including exposure history (date of exposure in high-risk areas such as Hubei, or the earliest and latest date of close contact with an infected case), source of infection (imported or local cases, suspected regions), date of key events (including onset, quarantine, and confirmation). The informed consent was obtained from each participant and this epidemiological study was approved by the Ethical Committee of Guangzhou CDC. In this study, we applied the county-level polygon from the National Geomatics Center of China (http://www.ngcc.cn/ngcc/), on which we generated a county-level layer containing information regarding latitudes and longitudes of COVID-19 cases in county layers of each county. We extracted the prevention and control measures in Guangzhou from January 20, 2020 to May 17, 2020 from the website of Guangzhou Government (http://www.gz.gov.cn/zt/qlyfdyyqfkyz/qktb/fygg/index.html).

To estimate the distribution of incubation period, an interval between the potential earliest date of contact of the transmission source and the potential earliest date of symptom onset,^[7]^ we included a total of 102 COVID-19 cases who meet the criterion below: COVID-19 infected individual that was symptomatic; patient that had a history of travel in Hubei within 14 days before symptom onset and stayed <7 days; patient that did not have a history of travel in Hubei but had a confirmed contact period with a COVID-19 confirmed case within 14 days before symptom onset. Patient with a history of staying in Hubei for >7 days, with unclear contact information or source of infection was excluded in this study.^[8]^ To estimate the serial interval, the time from symptom onset in a primary case (infector) to symptom onset in a secondary case (infectee),^[9]^ we included a total of 123 pairs of infector-infectee who meet the criterion below: there was scientifically linked between the primary case and secondary case; both infector and infectee were symptomatic. In our study, asymptomatic cases were analyzed as confirmed cases (see Supplementary Appendix A, Supplemental Digital Content, which illustrates cases definition).^[10]^

### 2.2 Statistical analysis

All analyses were done with R software, version 3.6.3 (R Foundation for Statistical Computing). The epidemic curves were constructed by date of confirmation, date of symptom onset and were sorted by cases type including imported or local cases. The continuous variables were expressed as means and standard deviations or medians and interquartile range (IQR), as deemed appropriate. The categorical variables were summarized as counts and percentages. We used ArcGIS, version 10.2.2, to plot the numbers of patients with COVID-19 cases on a county-level map of Guangzhou.

For each case, the date of exposure was between 2 possible dates, while the date of symptom onset was definite. Therefore, for the incubation period, such data were called single interval-censored data; for the serial interval, they were exact observations. To estimate the distribution of incubation period and serial interval, we fitted 3 common kinds of distribution (Weibull, gamma, and log-normal) to estimate the distribution of incubation period and serial interval by using coarseDataTools package and selected the best-fitted model by comparing the log-likelihood or Akaike information criterion values.^[11–13]^ The parameters including mean and standard deviation and specific quantiles (2.5th, 25th, 50th, 75th, and 97.5th percentiles) along with their bootstrapped 95% confidence intervals (CIs) were estimated for the best-fitted model. Further, we also estimated time-to-event distributions for COVID-19 cases, including symptom onset to quarantine and confirm by fitting 3 kinds of distribution above and selecting the best-fitted based on the minimum Akaike information criterion. To evaluate the effectiveness of public health interventions in Guangzhou by 3 periods: January 7 to 22 (no intervention), January 23 to February 23 (implemented intensive interventions), and February 24 to May 17 (the normalization mode of COVID-19 prevention and control).

Using the estimated distribution of the serial interval, we calculated the basic reproduction number (*R*_0_), which was defined as the expected number of additional cases that one case would generate, on average, over the course of its infectious period in an otherwise uninfected population. We calculated *R*_0_ between January 7 and January 23 by using maximum likelihood in *R*_0_ package which considered the impact of imported cases in Guangzhou. Besides, we calculated the effective reproduction number (*R*_*t*_), which was defined as the mean number of secondary cases generated by a typical primary case at time *t* considering the importations and local transmission, by using EpiEstim package.^[14]^

## 3 Result

### 3.1 Characteristics of patients with COVID-19 in Guangzhou

Guangzhou is located to the south of China, which is 986 km from Wuhan. Many people travel to Guangzhou from Hubei province because of the Spring Festival. According to the date of confirmation or symptom onset and the number of new cases, as shown in Fig. 1 (see Table S1, Supplemental Digital Content, which illustrates raw data), Guangzhou had experienced 2 COVID-19 outbreaks from January 21, 2020 to May 17, 2020 (see Supplementary Appendix B, Supplemental Digital Content, which illustrates 2 outbreaks of COVID-19).

As shown in Fig. 1A, we found that the epidemic curve of symptom onset by date was earlier than the epidemic curve by date of confirmation in the first outbreak (between January 7 and March 5), while these 2 curves were coincident in the second outbreak (between March 5 and May 2). The duration of the second outbreak lasted longer than that of the first outbreak (72 days vs 57 days). As illustrated in Fig. 1B and C, the outbreak in Guangzhou was mainly caused by imported cases. In the first outbreak, there was no apparent separation in time between imported cases and local cases. But the points worth noting that there was a significant separation in time between imported cases and local cases in the second outbreak indicating that there was a noteworthy COVID-19 outbreak of local cases occurred between April 6 and April 28.

We analyzed the proportion of COVID-19 cases (defined as the number of cases per million people) geographical locations across Guangzhou between January 7 and May 17 (see Fig. 1E and Table S2, Supplemental Digital Content, which illustrates the confirmed case rate per million people). The COVID-19 outbreak mainly occurred in urban districts in Guangzhou. Baiyun, Yuexiu, and Tianhe districts had the highest number of confirmed cases, with 181, 117, and 102 cases respectively; Panyu, Yuexiu, and Baiyun districts had the highest confirmed cases rate per million people, which respectively were 117.0, 96.7, and 65.1 per million respectively, while Conghua (4.6 per million) and Nansha (18.8 per million) districts had the lowest confirmed cases rate per million people.

As listed in Table 1, our analyses included a total of 745 confirmed cases between January 7 and May 17 in Guangzhou, China, including 496 (66.6%) primary cases and 249 (33.4%) infected close contacts. Among the confirmed cases, 421 (56.5%) were men and 324 (43.5%) were women, and the median age of the patients was 38.8 years (range, 0–90.0). Distribution characteristics of sex and age were exhibited (see Fig. S1, Supplemental Digital Content, and Table S3, Supplemental Digital Content, which illustrates age-distribution of different sex in cases). There are 157 confirmed cases (21.1%) from the Africa continent while 28 confirmed cases came from other regions worldwide only accounted for 3.7% among all cases. There were 275 (36.9%) were asymptomatic patients, 412 (55.3%) were the symptomatic case, 57 (7.7%) were severe cases, and 1 (0.1%) death case.

**Table 1:** Characteristics of laboratory-confirmed COVID-19 cases in Guangzhou from Jan 7 to May 17, 2020.

Characteristics	Total (n = 745) No./total no.(%)	Primary cases (n = 496) No./total no.(%)	Infected close contacts (n = 249) No./total no.(%)	*P* value
Gender, female	324/745 (43.5%)	206/496 (41.5%)	118/249 (47.4%)	.15
Age, Mean (SD)	38.8 (16.9)	38.7 (16.2)	39.0 (18.2)	.84
Region				.16
China	560/745 (75.2%)	383/496 (77.2%)	177/249 (71.1%)	
Africa	157/745 (21.1%)	100/496 (20.2%)	57/249 (22.9%)	
Asia (out of China)	5/745 (0.7%)	3/496 (0.6%)	2/249 (0.8%)	
Europe	12/745 (1.6%)	4/496 (0.8%)	8/249 (3.2%)	
North America	8/745 (1.1%)	4/496 (0.8%)	4/249 (1.6%)	
Oceania	1/745 (0.1%)	1/496 (0.2%)	0/249 (0.0%)	
South America	2/745 (0.3%)	1/496 (0.2%)	1/249 (0.4%)	
Cases type				<.01
Imported cases	438/745 (58.8%)	336/496 (67.7%)	102/249 (41.0%)	<.01
Imported from Hubei	214/438 (48.9%)	162/336 (48.2%)	52/102 (50.1%)	
Out of Hube (in China)	53/438 (12.1%)	29/336 (8.6%)	24/102 (24.4%)	
Imported from overseas	171/438 (39.0%)	145/336 (43.2%)	26/102 (25.5%)	
Local cases	307/745 (41.2%)	160/496 (32.3%)	147/249 (59.0%)	<.01
Associated with imported cases from Hubei	63/307 (20.5%)	19/160 (11.9%)	44/147 (29.9%)	
Associated with imported cases from overseas	208/307 (67.8%)	116/160 (72.5%)	92/147 (62.6%)	
Infected source cases unknown	36/307 (11.7%)	25/160 (15.6%)	11/147 (8.1%)	
Cluster	419/745 (56.2%)	195/496 (39.3%)	224/249 (90.0%)	<.01
Household cluster	342/419 (81.6%)	161/195 (82.6%)	181/224 (80.8%)	.64
Case of severity				.01
Asymptoms	275/745 (36.9%)	161/496 (32.5%)	114/249 (45.8%)	
Symptoms	412/745 (55.3%)	288/496 (58.0%)	124/249 (49.8%)	
Severe	57/745 (7.7%)	46/496 (9.3%)	11/249 (4.4%)	
Death	1/745 (0.1%)	1/496 (0.2%)	0	
Time from onset to diagnosis, mean (SD)	4.5 (4.4)	5.2 (4.7)	3.22 (3.3)	<.01
Time from onset to quarantine, mean (SD)	1.9 (5.2)	2.9 (5.4)	-0.29 (4.0)	<.01

Of the 745 confirmed cases, there were 438 (438/745, 58.8%) imported cases, which including 214 (214/438, 48.9%) that were imported from Hubei Province and 171 (171/438, 39.0%) that were imported from overseas (outside China), and 307 (307/745, 41.2%) local cases. We found that the proportion of imported cases and local cases in primary cases was different from that in infected close contacts (*P* <* *.01), Table 1 showed that the proportion of imported cases in primary cases was higher than that in infected close contacts (67.7% vs 41.0%) and the proportion of local cases in primary cases was lower than that in infected close contacts (32.3% vs 59.0%). Among both the imported cases or the local cases, the distributions of infection sources were both different between in primary cases and infected close contacts (*P* < .01). Among the cluster cases, the proportion of household clusters in cluster cases was especially high, accounting for 81.6% (342/419). Transmission of COVID-19 within families and close contacts accounts for the majority of epidemic growth.

### 3.2 The spectrum of COVID-19 cases from infected close contacts

We prospectively tracked 11,711 close contacts with COVID-19 patients, there were 249 infected close contacts (see Fig. S2, Supplemental Digital Content, which demonstrates the spectrum of cases from infected close contacts). Approximately 45.8%, 49.8%, and 4.4% of infected close contacts were asymptomatic, symptomatic, and severe, respectively. We evaluated the difference spectrum of COVID-19 cases between the primary cases and the infected close contacts. Compared with the primary cases, the proportion of asymptomatic cases (45.8% vs 32.5%, *P* < .01) were higher while the proportion of severe cases were lower (4.4% vs 9.3%, *P* < .01).

### 3.3 Estimation of the incubation period and serial interval

Based on 102 cases with a well-defined period of exposure and symptom onset, we estimated that the incubation period was the best fit with a log-normal distribution. The mean incubation period for COVID-19 was 6.5 days (95% CI, 5.6–7.4) with a standard error of 4.6 days and the median incubation period was 5.3 days (Table 2). We estimated that fewer than 2.5% of the infected people would show symptoms within 1.5 days of exposure, and symptom onset will occur within 18.4 days for 97.5% of infected people (see Table 2 and Table S4, Supplemental Digital Content, which illustrates parameter estimates for various parametric distributions of the incubation period and serial interval).

**Table 2:** Key time-to-event intervals for laboratory-confirmed COVID-19 cases by different periods, as of May 17, 2020.

				Percentiles					
	N	Mean (95% CI)	SD	2.5th	25th	50th	75th	97.5th	*P* value
Incubation period,^∗^ d	102	6.51 (5.62–7.40)	4.58	1.54	3.47	5.33	8.17	18.44	
Serial interval,^†^ d	123	5.95 (5.24–6.66)	3.99	0.84	3.02	5.08	7.95	15.92	
Onset-to-quarantined, d									
Whole period	392	5.36 (4.81–5.91)	5.53	0.7	2.1	3.72	6.62	19.79	–
Period 1	78	7.63 (6.93–8.33)	3.15	2.09	5.32	7.46	9.74	14.19	Ref
Period 2	197	4.49 (3.95–5.03)	3.9	0.78	2.04	3.39	5.62	14.73	<.0001
Period3	117	4.66 (3.57–5.75)	6.02	0.41	1.47	2.86	5.57	19.89	<.0001
Onset-to-confirmation, d									
Whole period	660	5.07 (4.67∼5.47)	5.19	0.67	2	3.54	6.27	18.64	–
Period 1	78	9.10 (8.29∼9.91)	3.63	2.62	6.45	8.93	11.55	16.59	Ref
Period 2	255	5.63 (5.19∼6.07)	3.62	0.9	2.97	4.87	7.47	14.61	<.0001
Period3	327	3.33 (2.98∼3.68)	3.21	0.49	1.39	2.4	4.15	11.75	<.0001

**Figure 2:**
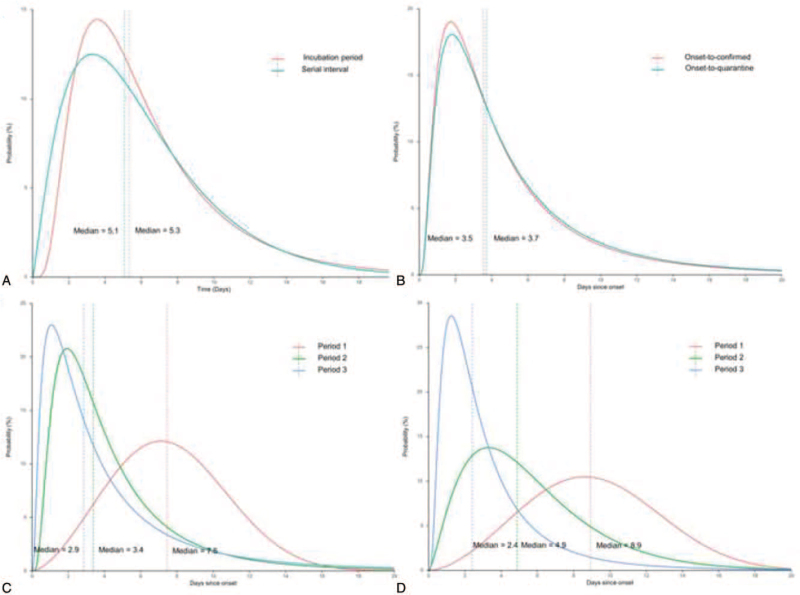
Key time-to-event distributions of COVID-19 cases in Guangzhou, China, 2020. The vertical dashed lines represent the medians of the best-fitted distribution. (A) Comparison between the best-fitting distributions of serial interval and incubation period distribution; (B) comparison between the best-fitting distributions of the onset-to-quarantine and of the onset-to-confirmed for whole period; (C) comparison between the best-fitting distributions of the onset-to-quarantine for 3 periods; (D) comparison between the best-fitting distributions of the onset-to-confirmed for 3 periods.

We analyzed the serial interval in 123 secondary cases and 64 corresponding primary cases (Table 2). The serial interval followed a gamma distribution with an estimated median of 5.1 days (percentiles of 2.5–97.5, 0.8–15.9 days). A comparison of the distribution of the incubation period and the serial interval was shown in Fig. 2A, which showed overlap between these 2 distributions.

### 3.4 Estimation of key time-to-event distributions of COVID-19

Fitting with log-normal distribution (see Table S4, Supplemental Digital Content, which illustrates parameter estimates for various parametric distributions of different periods of the time of onset-to-quarantined and time of onset-to-confirmation), we estimated the distribution of onset-to-quarantined in 392 cases with an estimated mean of 5.4 days (95% CI, 4.8–5.9), standard error of 5.5 days and median of 3.7 days (Table 2 and Fig. 2B). We estimated that fewer than 2.5% of COVID-19 cases would be quarantined within 0.7 days of symptom onset, and 97.5% of COVID-19 cases were quarantined within 19.8 days of symptom onset (Table 2).

Using data on the date of confirmation of 660 COVID-19 cases, we estimated a mean onset-to-confirmation time of 5.1 days (95% CI, 4.7–5.5) with standard error of 5.2 days and median of 3.5 days (Table 2 and Fig. 2B). We estimated that fewer than 2.5% of COVID-19 cases would be confirmed within 0.7 days of symptom onset, and to be confirmed within 18.6 days for 97.5% of COVID-19 cases.

We also estimated the onset-to-quarantined time and onset-to-confirmation time in these 3 periods (Table 2 and Fig. 2C, D). The estimated median of onset-to-quarantined time in Period 1 to 3 were 7.5, 3.4, and 2.9 days (the percentiles of 2.5–97.5, 2.1–14.2, 3.9–14.7, and 6.0–20.0 days) respectively and the median of onset-to-confirmation time in period 1 to 3 were 8.9, 4.9, and 2.4 days (the percentiles of 2.5–97.5, 2.6–16.6, 0.90–14.6, and 0.5–11.8 days). The onset-to-quarantined and onset-to-confirmation time in Period 2 and Period 3 were significantly shorter than those in Period 1 (*P* < .0001).

### 3.5 Prevention and control measures

The Spring Festival travel rush, a period of massive human movement in China, started in the early stage of COVID-19 outbreak in Guangzhou. On January 20, China CDC announced that one of the COVID-19 transmissions was a human-to-human transmission. At the same time, Guangzhou conducted temperature monitoring in traffic hubs.

On January 23, Guangzhou launched the first-level response to major public health emergencies. A series of strict interventions were implemented in Guangzhou since January 24, including the closure of public places, cancellation of public events, temperature monitoring, and disinfection in all public places, and isolation for people from Hubei and other epidemic areas in China.

The first-level response to emergencies was adjusted to second-level in Guangzhou on February 24. Guangzhou implemented the normalization mode of COVID-19 prevention and control, including isolation for people who had a history of foreign traveling in the 14 days and nucleic acid tests on them and their close contact (see Table S5, Supplemental Digital Content and Supplementary Appendix C, Supplemental Digital Content, which illustrates public health interventions of the COVID-19 outbreak in Guangzhou, China).

### 3.6 Evaluation of effects for prevention and control measures

Based on the analyzed serial interval, we estimated that the *R*_0_ to be 0.9 (95% CI, 0.5–1.4) for the cases with symptom onset between January 7 and January 23 by maximum likelihood, because we expected the proportion of infections would increase thereafter in Guangzhou.

**Figure 3:**
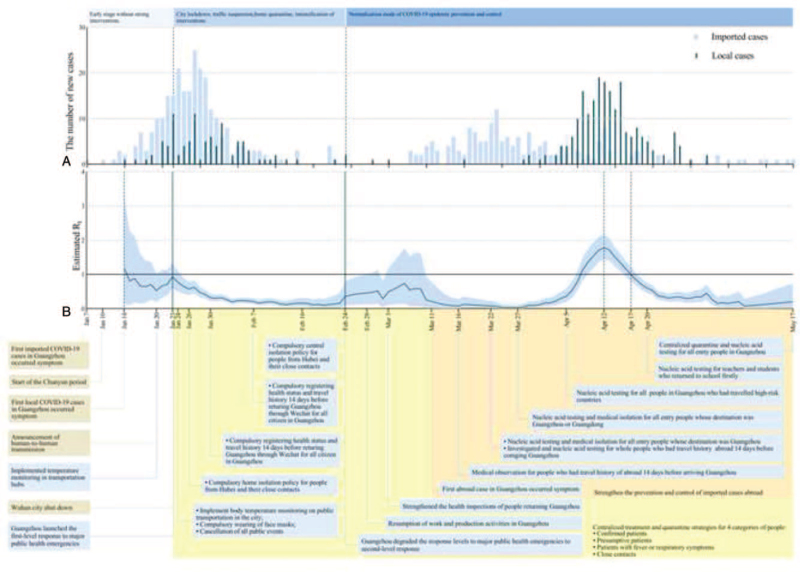
The effective reproduction number estimates based on laboratory-confirmed COVID-19 cases and public health control measures in Guangzhou. (A) The time distribution of imported and local cases by date of onset; (B) results were shown since January 14, calculated for the whole period (from January 14 to May 17) over 7-day moving average. The black horizontal line indicated *R*_*t*_ = 1, below which sustained transmission is unlikely so long as public health control measures were sustained, indicating that the outbreak is under control. The 95% credible intervals (CI) were presented as light bule shading. Daily estimates of *R*_*t*_ with 95% CrIs were shown in Supplementary Table 6, Supplemental Digital Content.

The epidemic curve according to the symptom onset date, estimation *R*_*t*_ and key interventions were shown in Fig. 3. We estimated *R*_*t*_ from January 14, 2020 to May 17, 2020, with a 7-day moving average, which was an index to evaluate the effect of prevention and control measures. *R*_*t*_ varied in period 1, gradually decreased in period 2 with the lowest point of 0.1 (95% CI, 0.0–0.3) on February 20. *R*_*t*_ was increasing since April 12 and reach a spike of 1.8 (95% CI, 1.4–2.2) in period 3. *R*_*t*_ decreased after April 12 and decreased to 1.0 on April 17 (see Table S6, Supplemental Digital Content, which illustrates the daily estimated values of *R*_*t*_).

## 4 Discussion

The COVID-19, a novel infectious disease caused by SARS-CoV2, is spreading rapidly around the world, while the epidemic in China has been effectively kept under control. The strategy of prevention and control had become normalized of which focus on domestic epidemics has shifted to overseas imported epidemics. This study analyzed the characteristic of 2 outbreaks and the intervention process in Guangzhou, which showed that taking strong measures against the source of infection, implementing preventive disinfection in epidemic sources and key places, improving protection ability of population, and evaluating the effect of intervention measures verified the effectiveness of containment strategies for epidemic control in megacities.^[15,16]^

We prospectively tracked 11,711 close contacts with COVID-19 patients, estimating the proportion of clinical severity among 249 infected close contacts to describe the spectrum of COVID-19 cases, which more effectively reflected the state of COVID-19 in the real world. We found that among 249 infected close contacts, 45.8% were asymptomatic, 49.8% were common symptoms, and 4.4% were severe. The spectrum of COVID-19 cases might be used to assess the burden of disease in the society and help adjust for more appropriate prevention and control strategies. The research suggested that currently severe affected countries such as the United States, Italy might be similar to the situation in China in the early stage of the epidemic. And the severity and rate of death were overestimated,^[17]^ which was mainly due to the ignorance of asymptomatic infections during the early stage of COVID-19 epidemic in China and other countries with severe epidemic like the United State and Italy. Further, the underlying cause of death of COVID-19 patients was unclear in the United State and Italy because the incidence of other diseases would also increase in the high-risk regions.^[18–20]^ Besides, the infected close contacts had a lower rate of severe and death compared with the primary COVID-19 cases, suggesting that the virulence of the virus may decrease with a passage. However, the results were derived from the analysis of the spectrum of COVID-19 cases in Guangzhou population. Whether the conclusion is valid on a larger population needs to be further confirmed by research.

From the analysis of the incubation period, the mean incubation period in Guangzhou was 6.5 days, and 97.5% of infected people would show symptoms within 18.4 days after SARS-CoV-2 infection. This further supported the understanding of COVID-19 as a disease with a fairly short incubation period (mean 4.9–7.4 days).^[7,21]^ The failure model analysis of a study assumed that there were 528.4 people with no symptoms found per 10,000 people monitored (the 99th percentile is 1092.7) among the infected population after 14 days of active surveillance (see Table S7, Supplemental Digital Content, which illustrates expected number of symptomatic SARS-CoV-2 infections that would be undetected during active monitoring, given varying monitoring durations, and risks for symptomatic infection after exposure).^[22]^ This study suggested that 10% of infected people may not be detected during the 14-day quarantine period, and therefore it is necessary to extend the quarantine period. If the quarantine period were extended to 21 days, only 1.7% of the infected will not be detected and the percentage would decrease to 0.2% if the quarantine period were extended to 28 days. It was recommended that a stricter 28-day quarantine period was appropriate, especially in cities or regions with high risk of infection.

From the analysis of infectivity, the *R*_0_ in the early stage in Guangzhou was 0.86 which suggested that the infectivity of COVID-19 in Guangzhou was limited since imported cases did not cause the local transmission in Guangzhou. Compared with other studies, Li et al^[7]^ calculated the *R*_0_ of COVID-19 in Wuhan before January 4 to be 2.2, and Zhao et al^[23]^ calculated the *R*_0_ between January 10 and February 24 to be 2.24 to 3.58, which were higher than those of Guangzhou. These studies came from the early stage of the COVID-19 epidemic when effective prevention and control measures were not taken, so the infectivity was relatively high. The estimation of *R*_0_ and the infectivity were underestimated for the result of missed diagnosis in the early stage of the epidemic. Asymptomatic infection was excluded in the management until February 5, 2020 which led to a largely missed diagnosis and the underestimated infectivity in the early stage of epidemic.^[24]^ Or the detection kits in the early stage were not sensitive and might produce false-negative results. The report showed that the detection rate of SARS-CoV-2 nucleic acid by real-time reverse transcriptase polymerase chain reaction was the amount for 30% to 50% of cases in the real world.^[25,26]^ Further, some studies had found that the viral load in the nasal cavity was higher than that in the throat, which indicated that nasal sampling was a more effective method.^[27]^ Throat swab, mainly used for sampling in Guangzhou, might have risk in standard operation and its specimens might be prone to produce false-negative results.^[28]^ The estimation of serial interval in Guangzhou was 5.9 days, suggesting that it took an average of 5.9 days for COVID-19 to spread from one case to the next in Guangzhou. Li et al^[7]^ calculated the serial interval of COVID-19 in Wuhan before January 4 to be 7.5 days, which was higher than those of Guangzhou, suggesting that the spread of SARS-CoV-2 in Guangzhou was 1.5 days faster than the early spread in Wuhan.^[29]^

In the early stage of the first outbreak, the number of imported cases in Guangzhou reached a peak from January 28 to January 30. With the development of the epidemic, the number of local cases had increased, but imported cases were still dominated. Guangzhou scaled up the first-level response to major public health emergencies, of which aim to prevent imported transmission.^[30]^ Based on the big data platform, the information collected was integrated with the records kept in the hospitals, CDCs, medical insurance agencies, public security and civil affairs authorities, telecommunication carriers, and other relevant units. Targeted nucleic acid tests and epidemiological studies were carried out on the 4 groups of people (i.e., confirmed cases, suspected cases, febrile patients who cannot rule out the possibility of infection, and close contacts), which helped to accurately and rapidly find close contacts and isolate them without delay, therefore effectively identifying and blocking the infection source.^[31]^ The lockdown of Wuhan and a series of prevention and control strategies conducted by Guangzhou, such as detecting the suspect COVID-19 cases, quarantining confirmed cases, tracking close contacts, successfully controlled the local spreading, when the last case that associated with Wuhan was confirmed and quarantined on March 5.^[32,33]^*R*_*t*_ showed a downward trend after January 23 and dropped to its minimum value of 0.1 on February 20, indicating that the prevention and control measures taken by Guangzhou had effectively controlled the local spread caused by the imported epidemic from Wuhan.

In the early stage of the second outbreak, people who were required to be quarantined were limited to some high-risk countries announced by China such as Japan, South Korea, and Italy. However, the number of COVID-19 cases announced by some countries may be different from the actual number. However, the severity of the epidemic may be underestimated,^[34]^ and imported cases from non-focused countries had not been detected in time. Guangzhou quarantined all people from overseas on March 22,^[35,36]^ and strengthened the investigation and management of entry people in Guangzhou that were assessed as high-risk on April 12.^[37]^ The overseas imported cases were effectively controlled before the onset of disease, which reduced the risk of cluster outbreaks and community transmission. The last infected case associated with overseas was diagnosed and quarantined on May 2, suggesting that the second outbreak was successfully controlled. So far, there had been no local recurrences of infection in Guangzhou since December 26, 2020. *R*_*t*_ showed an upward trend after March 27, peaked at 1.8 on April 12, then fell below 1 on April 17, and continued to decline, indicating that the prevention and control measures effectively controlled the local spread caused by the imported epidemic situation abroad. The successful control of the second outbreak provided lessons for cities or regions where the epidemic had rebounded globally. From key time-to-event distributions of COVID-19, the estimated means of onset-to-quarantined time in period 1 to 3 were 7.6, 4.5, and 4.7 days respectively and the estimated means of onset-to-confirmation time in period 1 to 3 were 9.1, 5.6, and 3.3 days. The results of this study showed that as the epidemic progressed, the response time from testing positive for COVID-19 to isolation and treatment of infected persons has shortened, suggesting the effectiveness of the blocking strategy implemented in Guangzhou.

Expanding the intensity and scope of COVID-19 nucleic acid testing could identify the most likely source of infection in Guangzhou. Over 514,000 people have been tested: including 197,000 teachers and students, 146,000 workers including health care, public health, community, public security, 139,000 (including 30,000 taxi drivers) in high-risk countries and regions in Guangzhou personnel, key places and population, and over 32,000 arrivals from overseas.^[38]^ The environment and food were sampled and conducted the nucleic acid test was to monitor the risk of transmission. Total coverage of 171,261 environmental and food sampling sites, all of them were negative for virus nucleic acid.^[39–41]^ According to the assessment, the possibility of SARS-CoV-2 contamination in the environment and food in various markets and restaurants in Guangzhou is very low, and the risk of virus transmission through the market environment and food pollution is low. A month after the outbreak of COVID-19 in China, a sample survey with more than 34,000 participants from the community to test for the antibody against the virus.^[42]^ The investigation found that the positive rate of antibody was 4.43% of the community population in Wuhan, 0.44% in other cities outside Wuhan of Hubei province, and only 2 antibody-positive cases were detected in more than 12,000 people in 6 provinces (including Guangzhou) outside Hubei province. According to the survey results, the population of Guangzhou has a very low rate of infection, which indicates that the epidemic situation control in Guangzhou has been successful and the spread of the epidemic was effectively prevented.^[43]^ As COVID-19 spread globally but with epidemiological characteristics becoming clearer, blocking strategies that carried out early detection of infected and close contact, early isolation treatment, and strict closed-loop management to reduce the chance of human transmission can effectively curb the large-scale epidemic spread brought by case delivery.^[44]^ Some studies had found that SARS-CoV-2 had an immune escape, which could coexist with its specific IgG antibody for up to 50 days.^[45,46]^ Only a small part of the population was immune and a specific drug has been absent for this virus so far. Although batches of COVID-19 vaccines had been successfully developed and approved, the safety and effectiveness of the vaccine need to be further verified in the period of <1 year from development to group immunity and facing new challenges with the rapid mutation of SARS-CoV-2. Some studies founded that the infectivity of SARS-CoV-2 had not weakened but had increased after rapid mutation. Whether an effective COVID-19 vaccine will be universally available in China or other countries within a year is unclear. In the context of the global super-pandemic, non-pharmaceutical interventions or containment strategies are currently the best choices for COVID-19 epidemic control.^[47–52]^

Through a detailed analysis of the effectiveness of prevention and control in Guangzhou, we had provided a successful model to control COVID-19 epidemic in a short period. However, this report had 3 limitations. First, this was a small prospective cohort study to evaluate the effectiveness of control measures. Second, some strict control measures, such as closed-loop management, centralized isolation of all high-risk people, may not apply to some countries and regions. Third, most of the cases were imported, they had a relatively long history of exposure to epidemic areas or had multiple exposures, it was difficult to accurately assess the time of infection for them.

## 5 Conclusion

The prospective and dynamic observation of COVID-19 cases and their close contacts that became infected provided a spectrum of COVID-19 cases from real world. The lockdown of Wuhan and closed-loop management of people arriving in Guangzhou were effective in halting the spread of SARS-CoV-2 in Guangzhou. The evaluation of the prevention and control strategies showed that series of multifaceted public health interventions had successfully controlled the epidemic of COVID-19.

## Acknowledgments

The authors thank Boqi Rao, Zhi Li, Yingyi Feng, Yujie Pan, Zeqin Huang, and Junyi Ye from Guangzhou Medical University (Guangzhou, China) for assisting with data collection. They thanked all medical staffs participating the prevention and control of COVID-19 and data collection, and thanked all patients assisting the epidemiological interviewing.

## Author contributions

**Conceptualization:** Hongjun Zhao, Jiachun Lu, Zhicong Yang.

**Data curation:** Hongjun Zhao, Wenhui Lun, Zongqiu Chen, Jingrong Li, Shunming Li, Ying Lu, Qing He.

**Formal analysis:** Wenhui Lun, Hongjun Zhao, Chun Mao, Jinbin Chen, Fuman Qiu.

**Funding acquisition:** Hongjun Zhao, Jiachun Lu.**Investigation:** Zhoubin Zhang, Zongqiu Chen, Jingrong Li, Shunming Li, Ying Lu, Qing He.

**Methodology:** Fuman Qiu.

**Project administration:** Zhicong Yang.

**Software:** Wenhui Lun, Chun Mao.

**Supervision:** Jiachun Lu, Zhicong Yang.

**Validation:** Jinbin Chen.

**Visualization:** Zhoubin Zhang, Jinbin Chen.

**Writing – original draft:** Hongjun Zhao, Wenhui Lun.

**Writing – review & editing:** Hongjun Zhao, Wenhui Lun, Xiaoxiao Lu, Jiachun Lu, Zhicong Yang.

## Supplementary Material

Supplemental Digital Content

## Supplementary Material

Supplemental Digital Content

## Supplementary Material

Supplemental Digital Content

## Supplementary Material

Supplemental Digital Content

## Supplementary Material

Supplemental Digital Content

## Supplementary Material

Supplemental Digital Content

## Supplementary Material

Supplemental Digital Content

## Supplementary Material

Supplemental Digital Content

## Supplementary Material

Supplemental Digital Content

## Supplementary Material

Supplemental Digital Content
