# Supplementary figures and images for: Transmission dynamics and successful control measures of SARS-CoV-2 in the mega-size city of Guangzhou, China
Source: Medicine (Baltimore). 2021 Dec 3;100(48):e27846. doi: 10.1097/MD.0000000000027846 (PMC9191374; doi:10.1097/MD.0000000000027846)

Figure S1. The sex (A) and age (B) distribution of 745 COVID-19 cases in Guangzhou.


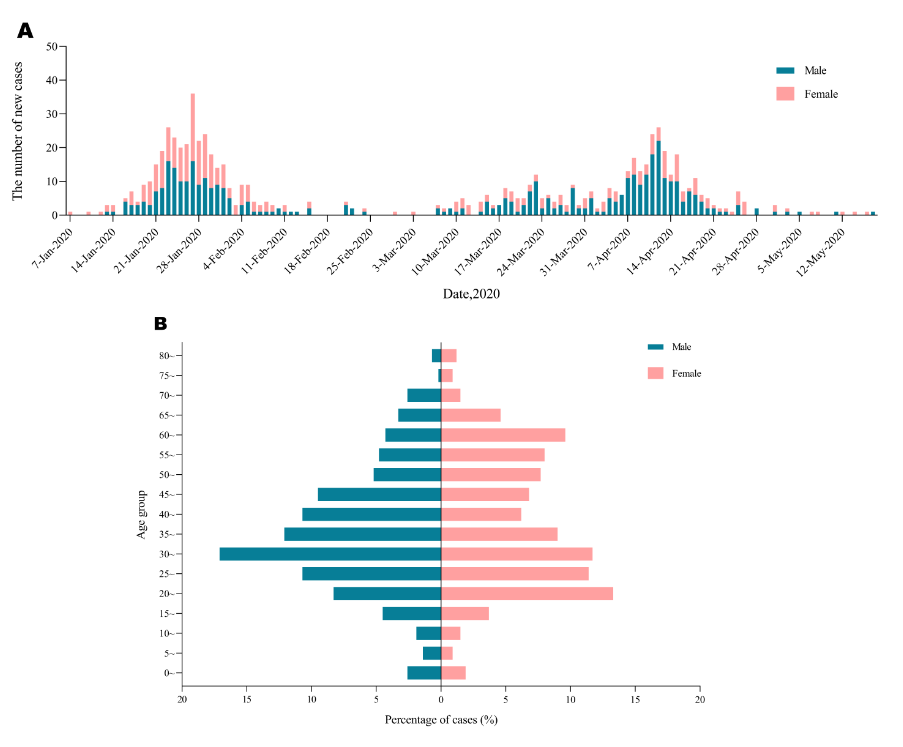

Supplement: Supplemental Digital Content [file medi-100-e27846-s001.doc]
