# Supplementary material for: Transmission dynamics and successful control measures of SARS-CoV-2 in the mega-size city of Guangzhou, China
Source: Medicine (Baltimore). 2021 Dec 3;100(48):e27846. doi: 10.1097/MD.0000000000027846 (PMC9191374; doi:10.1097/MD.0000000000027846)

Figure S2. Spectrum of COVID-19 cases. (A) Spectrum of total COVID-19 cases; (B) Spectrum of primary COVID-19 cases; (C) Spectrum of infected close contacts.


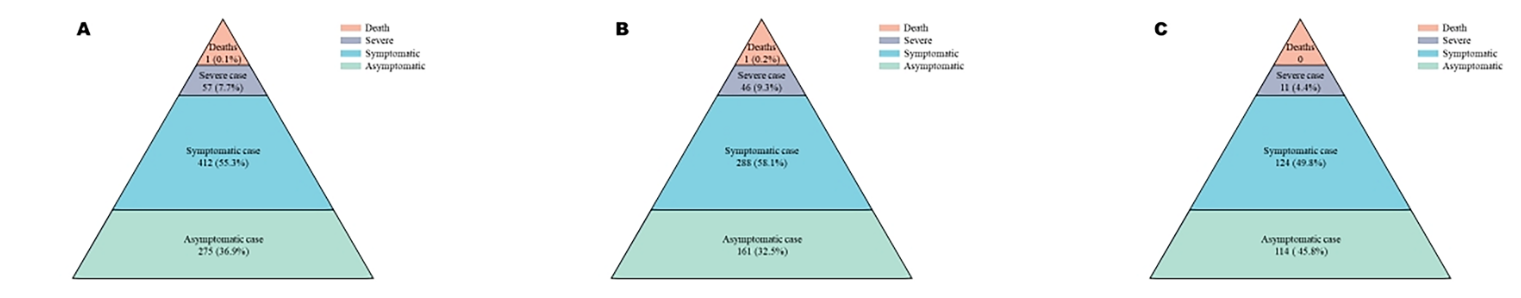

Supplement: Supplemental Digital Content [file medi-100-e27846-s002.doc]
