# Supplementary material for: Transmission dynamics and successful control measures of SARS-CoV-2 in the mega-size city of Guangzhou, China
Source: Medicine (Baltimore). 2021 Dec 3;100(48):e27846. doi: 10.1097/MD.0000000000027846 (PMC9191374; doi:10.1097/MD.0000000000027846)
Supplement: Supplemental Digital Content [file medi-100-e27846-s003.docx]

**Part A**

**Cases definition**

**The exposure and epidemiological history** were included as the following situations within 14 days before symptom onset: 1) had travelled to, or lived in Wuhan and surrounding areas, or other cities in China with case reports, or countries and regions abroad with severe epidemics; 2) had contact with COVID-19 confirmed cases; 3) had contact with patients who had a fever or other respiratory symptoms from Wuhan and surrounding areas, or other cities with case reports in China, or countries or regions with severe epidemics abroad; 4) **cluster outbreak**: two or more cases with fever or respiratory symptoms occurring within 14 days in the same confined space (such as home, office, school class, workshop, construction site, etc). **A close contact** was defined as an individual who had not taken effective protection (wearing mask) while being in close contact (within 1m) with suspected or confirmed cases 2 days before symptom onset or sample collection.^[1]^ **An imported case** was defined as an individual who had traveled to or resided in regions outside Guangzhou in the 2 weeks before symptom onset; otherwise, the individual was regarded as **a local case**.

**A suspected case** was defined as an individual who met any one of the following epidemiological histories and two or more clinical characteristics (1. had a fever or respiratory symptoms; 2. had radiologic imaging features of SARS-CoV-2 pneumonia; 3. had normal or reduced leucocyte count or reduced lymphocyte counts in the early stage of onset) or is an individual who had unclear epidemiological history but met three of the clinical characteristics. **A COVID-19 case** was defined as a suspected case that had subsequent laboratory confirmation with positive detection of SARS-CoV-2 nucleic acid by real-time reverse-transcriptase-polymerase-chain-reaction (RT-PCR) or positive detection of SARS-CoV-2 serum-specific IgM antibodies. **A primary COVID-19 case** is defined as a COVID-19 case who has travel to, living in Hubei or a COVID-19 case who has the earliest symptom onset date. **A COVID-19 case from close contacts** referred to a close contact who was laboratory-confirmed after having close contact with COVID-19 suspected or confirmed cases. **An asymptomatic case** was defined as an individual who was without any clinical symptom but with laboratory confirmation.^[1]^ In our study, asymptomatic cases were analyzed as confirmed cases.

**Reference**

1 National Health Commission of the People’s Republic of China. Prevention and control guideline on pneumonia infection with 2019 novel coronavirus (6th trial edn). 2020; published online March 7. http://www.nhc.gov.cn/xcs/zhengcwj/202003/4856d5b0458141fa9f376853224d41d7.shtml.

**Part B**

**Two outbreaks of COVID-19 in Guangzhou, China**

Guangzhou had experienced two outbreaks of COVID-19 during January 21^st^ to May 17^th^. The first COVID-19 epidemic was caused by the imported cases from Wuhan, Hubei and the second was caused by the imported cases from overseas, which led to a local spread. In the first epidemic, 262 cases were imported, resulting in 99 local infections, with a local infection rate of 27.4%. The earliest 3 COVID-19 cases, from different families, came from Wuhan on January 13^th^, 2020, and were confirmed and quarantined on January 22^nd^, January 24^th^, and January 26^th^ respectively. The number of imported cases in Guangzhou reached the peak from January 28^th^ to January 30^th^. With the development of the epidemic, the number of local cases had increased, but imported cases still dominated. The last case that associated with Wuhan was confirmed and quarantined on March 5^th^, indicating that the first epidemic was basically under control. During the second epidemic, 176 cases were imported while 208 were local infections, accounting for 54.2%. The first asymptomatic case entered the Guangzhou from Thailand on March 3^rd^ and was confirmed and quarantined on March 12^th^ while the earliest confirmed cases entered Guangzhou from Thailand on March 7^th^ and were confirmed on March 11^th^. The number of cases importing overseas reached a peak on March 23^rd^. As the epidemic went on, the number of local cases constantly increased and reached a peak on April 13^th^. The last infected case imported from overseas was diagnosed and quarantined on May 2^nd^, suggesting that the second epidemic was successfully controlled.

**Part C**

**Public health interventions of the COVID-19 outbreak in Guangzhou, China**

In the early stage of COVID-19 epidemic in Guangzhou, there were large population flow in Guangzhou but without strong interventions for COVID-19. Since January 10, the Chunyun, a period of massive human movement in China, started. On January 20, China CDC announced that one of the COVID-19 transmissions was human-to-human transmission. At the same time, Guangzhou conducted temperature monitoring in traffic hubs.

On January 23, Guangzhou launched the first-level response to major public health emergencies. A series of strict interventions were implemented in Guangzhou since January 24, including closure of public places, cancellation of public events, temperature monitoring and distinction in all public places, and home-isolation for people from Wuhan. Since January 26, all citizens were commanded to wear mask when they went out and people from Hubei were required to quarantine for 14 days. On January 30, all citizens in Guangzhou were required to record their travelling history and health status within 14 days through the “Suikang” application of WeChat, the most popular communication application in China. On February 7, all community or villages in Guangzhou were implemented closed-off management which set temperature monitor at the entrances and exits and only allow the residents living in this community or village to enter. Further, people arriving Guangzhou from the epidemic areas in China were required self-isolation or central-isolation for 14 days. Compulsory central quarantine and medical observation for all people arriving in Guangzhou from Hubei Province was implemented since February 13. The medical observation was removed if people did not show any symptoms after 14-day-observation.

The first-level response to emergencies was adjusted to second-level in Guangzhou on February 24. The number of new cases of COVID-19 decreased. Guangzhou implemented the normalization mode of COVID-19 prevention and control. Work and production activities gradually resumed in Guangzhou on February 28. On March 3, those who were still in high-risk areas in the country were not allowed to return to Guangzhou without authorization. People arriving Guangzhou from high-risk areas were commanded to centralized isolation for medical observation for 14. Public places were required to control the number of people entering and avoid crowd gathering. On March 16, in order to prevent the spread of the COVID-19 epidemic abroad, Guangzhou strengthened prevention and control measures, including port quarantining, medical observation, community screening, fever clinics and hospital treatment. Chinese tourists arriving Guangzhou who had a history of foreign travelling in the 14 days before arriving were transferred to destination by government and were required home-isolation or central-isolation for 14 days. On March 21, all tourists arriving Guangzhou who had a history of foreign travelling in the 14 days before arriving were required home-isolation or central-isolation for 14 days and to undergo compulsory nucleic acid testing of SARS-CoV-2. On March 22, Guangzhou launched a 14-day retrospective investigation for people with abroad travelling history. Anyone who came from abroad after March 8 and had a history of foreign travel within 14 days before coming to Guangzhou, regardless of whether it is a foreign or a Chinese, had to immediately undergo centralized or home isolation medical observation and declare their health status to the community workers before March 23. Those who had not undergone nucleic acid testing after entering would be subject to free throat swab sampling nucleic acid testing and antibody testing. All people who had been to high-risk countries and their close contacts were both investigated and tested since April 5. On April 20, Guangzhou conducted nucleic acid tests on 30,000 teachers and 167,000 junior and senior high school students who were ready to return to school. The second-level response to emergencies was adjusted to third-level in Guangzhou on May 8. On May 17, 2020, people entering from Guangzhou Port, regardless of destination, were commanded central quarantine and medical observation for 14 days and were required nucleic acid testing. Shopping malls, office buildings, supermarkets, hotels, restaurants, and other working and living places were re-opened. Primary and secondary schools, colleges and universities orderly resumed classes. All teachers and students must undergo nucleic acid testing before returning to school.
