# Supplementary material for: Transmission dynamics and successful control measures of SARS-CoV-2 in the mega-size city of Guangzhou, China
Source: Medicine (Baltimore). 2021 Dec 3;100(48):e27846. doi: 10.1097/MD.0000000000027846 (PMC9191374; doi:10.1097/MD.0000000000027846)
Supplement: Supplemental Digital Content [file medi-100-e27846-s004.docx]

Supplement Table 1. **Daily new cases of COVID-19 from January 7, 2020 to May 17, 2020 in Guangzhou, China**

| Date | By date of onset | | | By date of confirmation | | | By gender | |
| --- | --- | --- | --- | --- | --- | --- | --- | --- |
|  | Imported cases | Local cases | Total cases | Imported cases | Local cases | Total cases | Male cases | Female cases |
| 2020/1/7 | 1 | 0 | 1 | 0 | 0 | 0 | 0 | 1 |
| 2020/1/8 | 0 | 0 | 0 | 0 | 0 | 0 | 0 | 0 |
| 2020/1/9 | 0 | 0 | 0 | 0 | 0 | 0 | 0 | 0 |
| 2020/1/10 | 1 | 0 | 1 | 0 | 0 | 0 | 0 | 1 |
| 2020/1/11 | 0 | 0 | 0 | 0 | 0 | 0 | 0 | 0 |
| 2020/1/12 | 1 | 0 | 1 | 0 | 0 | 0 | 0 | 1 |
| 2020/1/13 | 3 | 0 | 3 | 0 | 0 | 0 | 1 | 2 |
| 2020/1/14 | 2 | 1 | 3 | 0 | 0 | 0 | 1 | 2 |
| 2020/1/15 | 0 | 0 | 0 | 0 | 0 | 0 | 0 | 0 |
| 2020/1/16 | 4 | 1 | 5 | 0 | 0 | 0 | 4 | 1 |
| 2020/1/17 | 7 | 0 | 7 | 0 | 0 | 0 | 3 | 4 |
| 2020/1/18 | 3 | 1 | 4 | 0 | 0 | 0 | 3 | 1 |
| 2020/1/19 | 7 | 2 | 9 | 0 | 0 | 0 | 4 | 5 |
| 2020/1/20 | 10 | 0 | 10 | 0 | 0 | 0 | 3 | 7 |
| 2020/1/21 | 10 | 5 | 15 | 2 | 0 | 2 | 7 | 8 |
| 2020/1/22 | 15 | 4 | 19 | 3 | 0 | 3 | 8 | 11 |
| 2020/1/23 | 15 | 11 | 26 | 2 | 0 | 2 | 16 | 10 |
| 2020/1/24 | 21 | 2 | 23 | 3 | 4 | 7 | 14 | 9 |
| 2020/1/25 | 16 | 4 | 20 | 16 | 3 | 19 | 10 | 10 |
| 2020/1/26 | 16 | 5 | 21 | 10 | 5 | 15 | 10 | 11 |
| 2020/1/27 | 25 | 11 | 36 | 18 | 5 | 23 | 16 | 20 |
| 2020/1/28 | 21 | 1 | 22 | 27 | 3 | 30 | 9 | 13 |
| 2020/1/29 | 19 | 5 | 24 | 34 | 10 | 44 | 11 | 13 |
| 2020/1/30 | 12 | 6 | 18 | 26 | 3 | 29 | 8 | 10 |
| 2020/1/31 | 10 | 4 | 14 | 16 | 6 | 22 | 9 | 5 |
| 2020/2/1 | 6 | 9 | 15 | 22 | 1 | 23 | 8 | 7 |
| 2020/2/2 | 8 | 0 | 8 | 19 | 2 | 21 | 5 | 3 |
| 2020/2/3 | 1 | 2 | 3 | 13 | 8 | 21 | 0 | 3 |
| 2020/2/4 | 4 | 5 | 9 | 9 | 5 | 14 | 3 | 6 |
| 2020/2/5 | 4 | 5 | 9 | 3 | 9 | 12 | 4 | 5 |
| 2020/2/6 | 1 | 3 | 4 | 6 | 11 | 17 | 1 | 3 |
| 2020/2/7 | 3 | 0 | 3 | 0 | 4 | 4 | 1 | 2 |
| 2020/2/8 | 3 | 1 | 4 | 6 | 3 | 9 | 1 | 3 |
| 2020/2/9 | 2 | 1 | 3 | 4 | 2 | 6 | 1 | 2 |
| 2020/2/10 | 1 | 1 | 2 | 4 | 1 | 5 | 2 | 0 |
| 2020/2/11 | 1 | 2 | 3 | 1 | 0 | 1 | 1 | 2 |
| 2020/2/12 | 1 | 0 | 1 | 1 | 4 | 5 | 1 | 0 |
| 2020/2/13 | 0 | 1 | 1 | 2 | 1 | 3 | 1 | 0 |
| 2020/2/14 | 0 | 0 | 0 | 5 | 2 | 7 | 0 | 0 |
| 2020/2/15 | 3 | 1 | 4 | 3 | 2 | 5 | 2 | 2 |
| 2020/2/16 | 0 | 0 | 0 | 0 | 0 | 0 | 0 | 0 |
| 2020/2/17 | 0 | 0 | 0 | 0 | 0 | 0 | 0 | 0 |
| 2020/2/18 | 0 | 0 | 0 | 0 | 0 | 0 | 0 | 0 |
| 2020/2/19 | 0 | 0 | 0 | 0 | 0 | 0 | 0 | 0 |
| 2020/2/20 | 0 | 0 | 0 | 1 | 0 | 1 | 0 | 0 |
| 2020/2/21 | 4 | 0 | 4 | 4 | 1 | 5 | 3 | 1 |
| 2020/2/22 | 1 | 1 | 2 | 1 | 1 | 2 | 2 | 0 |
| 2020/2/23 | 0 | 0 | 0 | 0 | 0 | 0 | 0 | 0 |
| 2020/2/24 | 0 | 2 | 2 | 1 | 0 | 1 | 1 | 1 |
| 2020/2/25 | 0 | 0 | 0 | 0 | 1 | 1 | 0 | 0 |
| 2020/2/26 | 0 | 0 | 0 | 0 | 0 | 0 | 0 | 0 |
| 2020/2/27 | 0 | 0 | 0 | 0 | 0 | 0 | 0 | 0 |
| 2020/2/28 | 0 | 0 | 0 | 0 | 0 | 0 | 0 | 0 |
| 2020/2/29 | 0 | 1 | 1 | 0 | 1 | 1 | 0 | 1 |
| 2020/3/1 | 0 | 0 | 0 | 0 | 0 | 0 | 0 | 0 |
| 2020/3/2 | 0 | 0 | 0 | 0 | 0 | 0 | 0 | 0 |
| 2020/3/3 | 0 | 1 | 1 | 0 | 0 | 0 | 0 | 1 |
| 2020/3/4 | 0 | 0 | 0 | 0 | 0 | 0 | 0 | 0 |
| 2020/3/5 | 0 | 0 | 0 | 0 | 1 | 1 | 0 | 0 |
| 2020/3/6 | 0 | 0 | 0 | 0 | 0 | 0 | 0 | 0 |
| 2020/3/7 | 3 | 0 | 3 | 0 | 0 | 0 | 2 | 1 |
| 2020/3/8 | 2 | 0 | 2 | 0 | 0 | 0 | 1 | 1 |
| 2020/3/9 | 2 | 0 | 2 | 0 | 0 | 0 | 2 | 0 |
| 2020/3/10 | 4 | 0 | 4 | 1 | 0 | 1 | 1 | 3 |
| 2020/3/11 | 5 | 0 | 5 | 2 | 0 | 2 | 2 | 3 |
| 2020/3/12 | 3 | 0 | 3 | 1 | 0 | 1 | 0 | 3 |
| 2020/3/13 | 0 | 0 | 0 | 0 | 0 | 0 | 0 | 0 |
| 2020/3/14 | 4 | 0 | 4 | 0 | 0 | 0 | 1 | 3 |
| 2020/3/15 | 6 | 0 | 6 | 1 | 0 | 1 | 4 | 2 |
| 2020/3/16 | 3 | 0 | 3 | 4 | 0 | 4 | 2 | 1 |
| 2020/3/17 | 2 | 1 | 3 | 4 | 0 | 4 | 3 | 0 |
| 2020/3/18 | 8 | 0 | 8 | 6 | 0 | 6 | 5 | 3 |
| 2020/3/19 | 7 | 0 | 7 | 4 | 0 | 4 | 4 | 3 |
| 2020/3/20 | 5 | 0 | 5 | 5 | 0 | 5 | 1 | 4 |
| 2020/3/21 | 5 | 0 | 5 | 3 | 1 | 4 | 3 | 2 |
| 2020/3/22 | 9 | 0 | 9 | 2 | 0 | 2 | 7 | 2 |
| 2020/3/23 | 12 | 0 | 12 | 12 | 0 | 12 | 10 | 2 |
| 2020/3/24 | 5 | 0 | 5 | 5 | 0 | 5 | 2 | 3 |
| 2020/3/25 | 6 | 0 | 6 | 9 | 0 | 9 | 5 | 1 |
| 2020/3/26 | 4 | 0 | 4 | 10 | 0 | 10 | 2 | 2 |
| 2020/3/27 | 6 | 0 | 6 | 8 | 0 | 8 | 3 | 3 |
| 2020/3/28 | 2 | 1 | 3 | 6 | 0 | 6 | 1 | 2 |
| 2020/3/29 | 7 | 2 | 9 | 7 | 0 | 7 | 8 | 1 |
| 2020/3/30 | 3 | 0 | 3 | 5 | 0 | 5 | 2 | 1 |
| 2020/3/31 | 4 | 1 | 5 | 9 | 0 | 9 | 2 | 3 |
| 2020/4/1 | 5 | 2 | 7 | 6 | 0 | 6 | 5 | 2 |
| 2020/4/2 | 2 | 0 | 2 | 4 | 1 | 5 | 1 | 1 |
| 2020/4/3 | 2 | 2 | 4 | 0 | 0 | 0 | 1 | 3 |
| 2020/4/4 | 4 | 4 | 8 | 2 | 3 | 5 | 5 | 3 |
| 2020/4/5 | 3 | 4 | 7 | 8 | 0 | 8 | 4 | 3 |
| 2020/4/6 | 0 | 6 | 6 | 2 | 1 | 3 | 6 | 0 |
| 2020/4/7 | 3 | 10 | 13 | 0 | 3 | 3 | 11 | 2 |
| 2020/4/8 | 1 | 16 | 17 | 2 | 3 | 5 | 12 | 5 |
| 2020/4/9 | 2 | 11 | 13 | 4 | 6 | 10 | 9 | 4 |
| 2020/4/10 | 1 | 14 | 15 | 1 | 13 | 14 | 12 | 3 |
| 2020/4/11 | 5 | 19 | 24 | 5 | 19 | 24 | 18 | 6 |
| 2020/4/12 | 8 | 18 | 26 | 5 | 27 | 32 | 22 | 4 |
| 2020/4/13 | 3 | 16 | 19 | 8 | 24 | 32 | 11 | 8 |
| 2020/4/14 | 0 | 12 | 12 | 0 | 17 | 17 | 10 | 2 |
| 2020/4/15 | 0 | 18 | 18 | 1 | 12 | 13 | 10 | 8 |
| 2020/4/16 | 0 | 7 | 7 | 2 | 19 | 21 | 4 | 3 |
| 2020/4/17 | 2 | 6 | 8 | 0 | 10 | 10 | 7 | 1 |
| 2020/4/18 | 3 | 8 | 11 | 2 | 7 | 9 | 6 | 5 |
| 2020/4/19 | 0 | 6 | 6 | 3 | 13 | 16 | 4 | 2 |
| 2020/4/20 | 0 | 5 | 5 | 0 | 5 | 5 | 2 | 3 |
| 2020/4/21 | 0 | 3 | 3 | 0 | 4 | 4 | 2 | 1 |
| 2020/4/22 | 2 | 0 | 2 | 2 | 3 | 5 | 1 | 1 |
| 2020/4/23 | 0 | 2 | 2 | 1 | 2 | 3 | 1 | 1 |
| 2020/4/24 | 1 | 0 | 1 | 0 | 1 | 1 | 0 | 1 |
| 2020/4/25 | 0 | 7 | 7 | 1 | 1 | 2 | 3 | 4 |
| 2020/4/26 | 0 | 4 | 4 | 1 | 3 | 4 | 0 | 4 |
| 2020/4/27 | 0 | 0 | 0 | 0 | 7 | 7 | 0 | 0 |
| 2020/4/28 | 1 | 1 | 2 | 0 | 0 | 0 | 2 | 0 |
| 2020/4/29 | 0 | 0 | 0 | 1 | 0 | 1 | 0 | 0 |
| 2020/4/30 | 0 | 0 | 0 | 0 | 1 | 1 | 0 | 0 |
| 2020/5/1 | 1 | 2 | 3 | 0 | 1 | 1 | 1 | 2 |
| 2020/5/2 | 0 | 0 | 0 | 0 | 1 | 1 | 0 | 0 |
| 2020/5/3 | 2 | 0 | 2 | 2 | 0 | 2 | 1 | 1 |
| 2020/5/4 | 0 | 0 | 0 | 1 | 0 | 1 | 0 | 0 |
| 2020/5/5 | 1 | 0 | 1 | 0 | 0 | 0 | 1 | 0 |
| 2020/5/6 | 0 | 0 | 0 | 1 | 0 | 1 | 0 | 0 |
| 2020/5/7 | 1 | 0 | 1 | 0 | 0 | 0 | 0 | 1 |
| 2020/5/8 | 1 | 0 | 1 | 0 | 0 | 0 | 0 | 1 |
| 2020/5/9 | 0 | 0 | 0 | 1 | 0 | 1 | 0 | 0 |
| 2020/5/10 | 0 | 0 | 0 | 0 | 0 | 0 | 0 | 0 |
| 2020/5/11 | 1 | 0 | 1 | 0 | 0 | 0 | 1 | 0 |
| 2020/5/12 | 1 | 0 | 1 | 1 | 0 | 1 | 0 | 1 |
| 2020/5/13 | 0 | 0 | 0 | 1 | 0 | 1 | 0 | 0 |
| 2020/5/14 | 1 | 0 | 1 | 1 | 0 | 1 | 0 | 1 |
| 2020/5/15 | 0 | 0 | 0 | 1 | 0 | 1 | 0 | 0 |
| 2020/5/16 | 1 | 0 | 1 | 1 | 0 | 1 | 0 | 1 |
| 2020/5/17 | 1 | 0 | 1 | 1 | 0 | 1 | 1 | 0 |
