# Supplementary material for: Transmission dynamics and successful control measures of SARS-CoV-2 in the mega-size city of Guangzhou, China
Source: Medicine (Baltimore). 2021 Dec 3;100(48):e27846. doi: 10.1097/MD.0000000000027846 (PMC9191374; doi:10.1097/MD.0000000000027846)
Supplement: Supplemental Digital Content [file medi-100-e27846-s005.docx]

Supplementary Table 2. **The COVID-19 confirmed case rate per million people in Guangzhou**

| District | Confirmed case (n) | Population (ten thousand) | Rate (per million) |
| --- | --- | --- | --- |
| Baiyun | 181 | 277.96 | 65.1 |
| Conghua | 3 | 64.95 | 4.6 |
| Haizhu | 80 | 172.42 | 46.4 |
| Huadu | 52 | 110.72 | 47.0 |
| Huangpu | 34 | 79.61 | 42.7 |
| Liwan | 46 | 101.2 | 45.5 |
| Nansha | 15 | 79.61 | 18.8 |
| Panyu | 76 | 64.95 | 117.0 |
| Tianhe | 102 | 178.85 | 57.0 |
| Yuexiu | 117 | 120.97 | 96.7 |
| Zengcheng | 36 | 126.01 | 28.6 |
