# Supplementary material for: Transmission dynamics and successful control measures of SARS-CoV-2 in the mega-size city of Guangzhou, China
Source: Medicine (Baltimore). 2021 Dec 3;100(48):e27846. doi: 10.1097/MD.0000000000027846 (PMC9191374; doi:10.1097/MD.0000000000027846)
Supplement: Supplemental Digital Content [file medi-100-e27846-s006.docx]

Supplement Table 3. **Age-distribution of different gender in COVID-19 cases in Guangzhou, China**

| Age group | Gender - n (%) | | Total - n (%) |
| --- | --- | --- | --- |
|  | Male | Female |  |
| 0~4 | 11 (2.6) | 6 (1.9) | 17 (2.3) |
| 5~9 | 6 (1.4) | 3 (0.9) | 9 (1.2) |
| 10~14 | 8 (1.9) | 5 (1.5) | 13 (1.7) |
| 15~19 | 19 (4.5) | 12 (3.7) | 31 (4.2) |
| 20~24 | 35 (8.3) | 43 (13.3) | 78 (10.5) |
| 25~29 | 45 (10.7) | 37 (11.4) | 82 (11) |
| 30~34 | 72 (17.1) | 38 (11.7) | 110 (14.8) |
| 35~39 | 51 (12.1) | 29 (9) | 80 (10.7) |
| 40~44 | 45 (10.7) | 20 (6.2) | 65 (8.7) |
| 45~49 | 40 (9.5) | 22 (6.8) | 62 (8.3) |
| 50~54 | 22 (5.2) | 25 (7.7) | 47 (6.3) |
| 55~59 | 20 (4.8) | 26 (8) | 46 (6.2) |
| 60~64 | 18 (4.3) | 31 (9.6) | 49 (6.6) |
| 65~69 | 14 (3.3) | 15 (4.6) | 29 (3.9) |
| 70~74 | 11 (2.6) | 5 (1.5) | 16 (2.1) |
| 75~79 | 1 (0.2) | 3 (0.9) | 4 (0.5) |
| ≥80 | 3 (0.7) | 4 (1.2) | 7 (0.9) |
