# Supplementary material for: Transmission dynamics and successful control measures of SARS-CoV-2 in the mega-size city of Guangzhou, China
Source: Medicine (Baltimore). 2021 Dec 3;100(48):e27846. doi: 10.1097/MD.0000000000027846 (PMC9191374; doi:10.1097/MD.0000000000027846)
Supplement: Supplemental Digital Content [file medi-100-e27846-s007.docx]

Supplement Table 4. **Parameter Estimates for Various Parametric Distributions of the Incubation Period, Serial Interval, the time of Onset-to-quarantined and time of Onset-to-confirmation of COVID-19 cases in Guangzhou, China**

|  |  | Lognormal | | | Gamma | | | Weibull | | |
| --- | --- | --- | --- | --- | --- | --- | --- | --- | --- | --- |
|  |  | meanlog (95% CI) | sdlog (95% CI) | Log Likelihood or AIC* | shape (95% CI) | rate (95% CI) | Log Likelihood or AIC* | shape (95% CI) | scale (95% CI) | Log Likelihood or AIC* |
| Incubation Period (n = 102) | | 1.672 (1.530 ~ 1.818) | 0.634 (0.533 ~ 0.721) | -129.65^*^ | 2.548 (2.004 ~ 3.494) | 2.535 (1.761 ~ 3.337) | -131.75 | 1.615 (1.395 ~ 1.985) | 7.214 (6.307 ~ 8.208) | -130.00 |
| Serial Interval (n = 123) | | 1.542 (1.531 ~ 1.554) | 0.726 (0.718 ~ 0.734) | 653.75 | 2.224 (2.177 ~ 2.271) | 0.374 (0.365 ~ 0.383) | 652.34^*^ | 1.532 (1.514 ~ 1.550) | 6.645 (6.572 ~ 6.718) | 656.97 |
| Onset-to-quarantined | Whole period (n = 392) | 1.314 (1.311 ~ 1.319) | 0.852 (0.849 ~ 0.855) | 2021.97^*^ | 1.631 (1.620 ~ 1.641) | 0.313 (0.310 ~ 0.315) | 2034.03 | 1.287 (1.282 ~ 1.292) | 5.668 (5.645 ~ 5.691) | 2044.72 |
|  | Period 1 (n = 78) | 1.936 (1.924 ~ 1.948) | 0.480 (0.471 ~ 0.488) | 412.79 | 5.274 (5.092 ~ 5.456) | 0.690 (0.665 ~ 0.715) | 402.55 | 2.604 (2.555 ~ 2.654) | 8.592 (8.505 ~ 8.680) | 400.06^*^ |
|  | Period 2 (n = 197) | 1.225 (1.217 ~ 1.232) | 0.753 (0.747 ~ 0.758) | 933.70^*^ | 1.944 (1.919 ~ 1.969) | 0432 (0.426 ~ 0.439) | 947.90 | 1.383 (1.372 ~ 1.393) | 4.965 (4.927 ~ 5.003) | 958.60 |
|  | Period 3 (n = 117) | 1.053 (1.036 ~ 1.070) | 0.993 (0.981 ~ 1.005) | 580.83^*^ | 1.102 (1.079 ~ 1.125) | 0.229 (0.223 ~ 0.235) | 604.96 | 0.994 (0.981 ~ 1.006) | 4.796 (4.711 ~ 4.882) | 605.63 |
| Onset-to-confirmation | Whole period (n = 660) | 1.265 (1.263 ~ 1.268) | 0.847 (0.845 ~ 0.849) | 3328.17^*^ | 1.598 (1.592 ~ 1.604) | 0.320 (0.318 ~ 0.321) | 3372.37 | 1.257 (1.254 ~ 1.260) | 5.411 (5.397 ~ 5.425) | 3394.39 |
|  | Period 1 (n=78) | 2.117 (2.106 ~ 2.128) | 0.447 (0.439 ~0.455) | 429.99 | 5.728 (5.530 ~ 5.925) | 0.630 (0.607 ~ 0.653) | 424.05 | 2.705 (2.652 ~ 2.757) | 10.233 (10.132 ~ 10.333) | 422.96^*^ |
|  | Period 2 (n = 255) | 1.504 (1.499 ~ 1.510) | 0.693 (0.689 ~ 0.697) | 1308.00 | 2.422 (2.397 ~ 2.447) | 0.432 (0.427 ~ 0.436) | 1304.08^*^ | 1.619 (1.608 ~ 1.630) | 6.298 (6.262 ~ 6.334) | 1312.884 |
|  | Period 3 (n = 327) | 0.876 (0.871 ~ 0.880) | 0.810 (0.807 ~ 0.814) | 1366.97^*^ | 1.421 (1.401 ~ 1.432) | 0.400 (0.397 ~ 0.404) | 1464.33 | 1.092 (1.087 ~ 1.096) | 3.698 (3.676 ~ 3.720) | 1481.67 |

* Log-likelihood and AIC (Akaike information criterion) are the criterion of statistical model goodness of fit. We selected the distribution of that the value of log- likelihood or AIC was the smallest to analyze the incubation period, serial interval the time of onset-to-quarantined and time of onset-to-confirmation of COVID-19 cases in Guangzhou, China.
