# Supplementary material for: Transmission dynamics and successful control measures of SARS-CoV-2 in the mega-size city of Guangzhou, China
Source: Medicine (Baltimore). 2021 Dec 3;100(48):e27846. doi: 10.1097/MD.0000000000027846 (PMC9191374; doi:10.1097/MD.0000000000027846)
Supplement: Supplemental Digital Content [file medi-100-e27846-s008.docx]

Supplementary Table 5. **Public health interventions of the COVID-19 outbreak in Guangzhou, China**

| Control the source of infection | Cut the transmission routes | Prevent new infestions | Starting date |
| --- | --- | --- | --- |
| **Early-stage without strong interventions** | | | |
|  | Investigation of the trade of wild animals |  | 2020/1/20 |
|  | Temperature monitoring in traffic hubs |  |  |
| Designation of hospitals for COVID-19 treatment |  | Recommendation of personal hygiene (such as washing hands) | 2020/1/22 |
| **Intensification of interventions** | | | |
| Surveillance for people from Wuhan after January 10 | Closure of public places of amusement |  | 2020/1/24 |
|  | Cancellation of gatherings and public events |  |  |
| Self-isolation or central-isolation for people from Hubei | Temperature monitoring and disinfection in all public places | Compulsory of wearing mask | 2020/1/26 |
|  |  | Recommendation for all people to record health status in an application | 2020/1/30 |
| Self-isolation or central-isolation for people from epidemic areas in China | Closure of cultural and entertainment gathering places |  | 2020/2/7 |
|  | Implement of closed-off management in communities and villages |  |  |
| Compulsory central-isolation for people from Hubei |  |  | 2020/2/13 |
| **Normalization mode of COVID-19 epidemic prevention and control** | | | |
| Compulsory central-isolation for people from epidemic areas in China | Reopening of communities and villages with temperature monitoring |  | 2020/3/3 |
|  | Reopening of commercial public places under the control the number of people entering |  |  |
| Self-isolation or central-isolation for Chinese tourists from oversea |  |  | 2020/3/16 |
| Self-isolation or central-isolation for tourists from oversea |  |  | 2020/3/21 |
| Self-isolation or central-isolation and nucleic acid test for people from oversea after March 8 |  |  | 2020/3/22 |
| Compulsory central-isolation and nucleic acid test for people from oversea |  |  | 2020/3/27 |
| Nucleic acid test for people who had been to high-risk countries and their close contact |  |  | 2020/4/5 |
| Nucleic acid test for teachers and students who returned to school |  |  | 2020/4/20 |
|  | Reopening of all public places |  | 2020/5/17 |
