# Supplementary material for: Transmission dynamics and successful control measures of SARS-CoV-2 in the mega-size city of Guangzhou, China
Source: Medicine (Baltimore). 2021 Dec 3;100(48):e27846. doi: 10.1097/MD.0000000000027846 (PMC9191374; doi:10.1097/MD.0000000000027846)
Supplement: Supplemental Digital Content [file medi-100-e27846-s009.docx]

Supplementary Table 6. **Estimation of the effective reproduction number (R_t_) for laboratory-confirmed COVID-19 cases in Guangzhou from January 14 to May 17, 2020**

| Date | Mean | 95% CI of mean | Sd | Median |
| --- | --- | --- | --- | --- |
| 1/14 | 1.20 | 0.14~3.33 | 0.85 | 1.00 |
| 1/15 | 0.81 | 0.10~2.26 | 0.57 | 0.68 |
| 1/16 | 0.88 | 0.18~2.11 | 0.51 | 0.78 |
| 1/17 | 0.66 | 0.14~1.59 | 0.38 | 0.59 |
| 1/18 | 0.64 | 0.17~1.41 | 0.32 | 0.59 |
| 1/19 | 0.70 | 0.26~1.37 | 0.29 | 0.66 |
| 1/20 | 0.52 | 0.19~1.02 | 0.21 | 0.50 |
| 1/21 | 0.66 | 0.32~1.13 | 0.21 | 0.64 |
| 1/22 | 0.71 | 0.39~1.13 | 0.19 | 0.69 |
| 1/23 | 0.93 | 0.60~1.34 | 0.19 | 0.92 |
| 1/24 | 0.76 | 0.50~1.09 | 0.15 | 0.75 |
| 1/25 | 0.65 | 0.44~0.91 | 0.12 | 0.65 |
| 1/26 | 0.57 | 0.39~0.79 | 0.10 | 0.57 |
| 1/27 | 0.63 | 0.45~0.83 | 0.10 | 0.62 |
| 1/28 | 0.48 | 0.34~0.64 | 0.08 | 0.47 |
| 1/29 | 0.41 | 0.30~0.55 | 0.07 | 0.41 |
| 1/30 | 0.32 | 0.22~0.43 | 0.05 | 0.31 |
| 1/31 | 0.30 | 0.21~0.41 | 0.05 | 0.30 |
| 2/1 | 0.32 | 0.23~0.42 | 0.05 | 0.32 |
| 2/2 | 0.27 | 0.19~0.36 | 0.04 | 0.27 |
| 2/3 | 0.20 | 0.13~0.28 | 0.04 | 0.20 |
| 2/4 | 0.23 | 0.16~0.31 | 0.04 | 0.23 |
| 2/5 | 0.24 | 0.16~0.32 | 0.04 | 0.23 |
| 2/6 | 0.23 | 0.15~0.32 | 0.04 | 0.22 |
| 2/7 | 0.21 | 0.14~0.30 | 0.04 | 0.21 |
| 2/8 | 0.16 | 0.09~0.24 | 0.04 | 0.15 |
| 2/9 | 0.19 | 0.11~0.28 | 0.04 | 0.18 |
| 2/10 | 0.20 | 0.11~0.30 | 0.05 | 0.19 |
| 2/11 | 0.18 | 0.10~0.29 | 0.05 | 0.18 |
| 2/12 | 0.13 | 0.06~0.23 | 0.04 | 0.13 |
| 2/13 | 0.12 | 0.05~0.22 | 0.04 | 0.11 |
| 2/14 | 0.14 | 0.06~0.26 | 0.05 | 0.13 |
| 2/15 | 0.16 | 0.06~0.30 | 0.06 | 0.15 |
| 2/16 | 0.16 | 0.06~0.31 | 0.06 | 0.15 |
| 2/17 | 0.15 | 0.05~0.31 | 0.07 | 0.14 |
| 2/18 | 0.11 | 0.02~0.26 | 0.06 | 0.10 |
| 2/19 | 0.13 | 0.03~0.30 | 0.07 | 0.11 |
| 2/20 | 0.10 | 0.01~0.28 | 0.07 | 0.08 |
| 2/21 | 0.12 | 0.01~0.33 | 0.08 | 0.10 |
| 2/22 | 0.14 | 0.02~0.39 | 0.10 | 0.12 |
| 2/23 | 0.16 | 0.02~0.44 | 0.11 | 0.13 |
| 2/24 | 0.36 | 0.10~0.78 | 0.18 | 0.33 |
| 2/25 | 0.40 | 0.11~0.87 | 0.20 | 0.36 |
| 2/26 | 0.42 | 0.12~0.93 | 0.21 | 0.39 |
| 2/27 | 0.44 | 0.12~0.97 | 0.22 | 0.41 |
| 2/28 | 0.46 | 0.12~1.00 | 0.23 | 0.42 |
| 2/29 | 0.47 | 0.13~1.04 | 0.24 | 0.44 |
| 3/1 | 0.52 | 0.14~1.13 | 0.26 | 0.47 |
| 3/2 | 0.29 | 0.03~0.80 | 0.20 | 0.24 |
| 3/3 | 0.49 | 0.10~1.18 | 0.28 | 0.44 |
| 3/4 | 0.57 | 0.12~1.36 | 0.33 | 0.50 |
| 3/5 | 0.65 | 0.13~1.56 | 0.37 | 0.58 |
| 3/6 | 0.73 | 0.15~1.77 | 0.42 | 0.65 |
| 3/7 | 0.55 | 0.07~1.52 | 0.39 | 0.46 |
| 3/8 | 0.59 | 0.07~1.64 | 0.42 | 0.49 |
| 3/9 | 0.57 | 0.07~1.60 | 0.41 | 0.48 |
| 3/10 | 0.26 | 0.01~0.94 | 0.26 | 0.18 |
| 3/11 | 0.21 | 0.01~0.79 | 0.21 | 0.15 |
| 3/12 | 0.17 | 0.00~0.62 | 0.17 | 0.12 |
| 3/13 | 0.13 | 0.00~0.48 | 0.13 | 0.09 |
| 3/14 | 0.10 | 0.00~0.38 | 0.10 | 0.07 |
| 3/15 | 0.09 | 0.00~0.32 | 0.09 | 0.06 |
| 3/16 | 0.07 | 0.00~0.27 | 0.07 | 0.05 |
| 3/17 | 0.13 | 0.02~0.36 | 0.09 | 0.11 |
| 3/18 | 0.12 | 0.01~0.32 | 0.08 | 0.10 |
| 3/19 | 0.11 | 0.01~0.29 | 0.07 | 0.09 |
| 3/20 | 0.10 | 0.01~0.27 | 0.07 | 0.08 |
| 3/21 | 0.09 | 0.01~0.24 | 0.06 | 0.07 |
| 3/22 | 0.08 | 0.01~0.22 | 0.06 | 0.07 |
| 3/23 | 0.07 | 0.01~0.20 | 0.05 | 0.06 |
| 3/24 | 0.03 | 0.00~0.12 | 0.03 | 0.02 |
| 3/25 | 0.03 | 0.00~0.11 | 0.03 | 0.02 |
| 3/26 | 0.03 | 0.00~0.10 | 0.03 | 0.02 |
| 3/27 | 0.03 | 0.00~0.09 | 0.03 | 0.02 |
| 3/28 | 0.05 | 0.01~0.14 | 0.03 | 0.04 |
| 3/29 | 0.09 | 0.03~0.21 | 0.05 | 0.09 |
| 3/30 | 0.09 | 0.03~0.20 | 0.05 | 0.08 |
| 3/31 | 0.11 | 0.04~0.24 | 0.05 | 0.11 |
| 4/1 | 0.16 | 0.07~0.31 | 0.06 | 0.16 |
| 4/2 | 0.17 | 0.07~0.31 | 0.06 | 0.16 |
| 4/3 | 0.22 | 0.10~0.39 | 0.07 | 0.21 |
| 4/4 | 0.30 | 0.16~0.50 | 0.09 | 0.29 |
| 4/5 | 0.36 | 0.20~0.57 | 0.10 | 0.35 |
| 4/6 | 0.52 | 0.32~0.78 | 0.12 | 0.51 |
| 4/7 | 0.76 | 0.51~1.07 | 0.14 | 0.76 |
| 4/8 | 1.12 | 0.81~1.48 | 0.17 | 1.11 |
| 4/9 | 1.35 | 1.02~1.74 | 0.18 | 1.35 |
| 4/10 | 1.54 | 1.19~1.93 | 0.19 | 1.53 |
| 4/11 | 1.71 | 1.35~2.10 | 0.19 | 1.70 |
| 4/12 | 1.77 | 1.44~2.15 | 0.18 | 1.77 |
| 4/13 | 1.71 | 1.40~2.05 | 0.17 | 1.70 |
| 4/14 | 1.50 | 1.23~1.80 | 0.15 | 1.50 |
| 4/15 | 1.34 | 1.10~1.61 | 0.13 | 1.34 |
| 4/16 | 1.16 | 0.95~1.40 | 0.11 | 1.16 |
| 4/17 | 0.99 | 0.81~1.20 | 0.10 | 0.99 |
| 4/18 | 0.84 | 0.67~1.02 | 0.09 | 0.83 |
| 4/19 | 0.70 | 0.55~0.87 | 0.08 | 0.70 |
| 4/20 | 0.60 | 0.46~0.75 | 0.08 | 0.59 |
| 4/21 | 0.53 | 0.40~0.68 | 0.07 | 0.53 |
| 4/22 | 0.37 | 0.26~0.50 | 0.06 | 0.37 |
| 4/23 | 0.35 | 0.24~0.48 | 0.06 | 0.34 |
| 4/24 | 0.31 | 0.20~0.44 | 0.06 | 0.30 |
| 4/25 | 0.33 | 0.21~0.48 | 0.07 | 0.33 |
| 4/26 | 0.34 | 0.21~0.50 | 0.07 | 0.34 |
| 4/27 | 0.30 | 0.18~0.46 | 0.07 | 0.29 |
| 4/28 | 0.30 | 0.17~0.47 | 0.08 | 0.30 |
| 4/29 | 0.34 | 0.19~0.54 | 0.09 | 0.34 |
| 4/30 | 0.34 | 0.18~0.55 | 0.09 | 0.33 |
| 5/1 | 0.45 | 0.25~0.70 | 0.12 | 0.44 |
| 5/2 | 0.27 | 0.12~0.49 | 0.10 | 0.26 |
| 5/3 | 0.15 | 0.04~0.34 | 0.08 | 0.14 |
| 5/4 | 0.18 | 0.05~0.39 | 0.09 | 0.16 |
| 5/5 | 0.15 | 0.03~0.37 | 0.09 | 0.14 |
| 5/6 | 0.18 | 0.04~0.43 | 0.10 | 0.16 |
| 5/7 | 0.20 | 0.04~0.49 | 0.12 | 0.18 |
| 5/8 | 0.08 | 0.00~0.29 | 0.08 | 0.05 |
| 5/9 | 0.09 | 0.00~0.32 | 0.09 | 0.06 |
| 5/10 | 0.10 | 0.00~0.37 | 0.10 | 0.07 |
| 5/11 | 0.11 | 0.00~0.41 | 0.11 | 0.08 |
| 5/12 | 0.13 | 0.00~0.47 | 0.13 | 0.09 |
| 5/13 | 0.14 | 0.00~0.53 | 0.14 | 0.10 |
| 5/14 | 0.16 | 0.00~0.58 | 0.16 | 0.11 |
| 5/15 | 0.17 | 0.00~0.63 | 0.17 | 0.12 |
| 5/16 | 0.18 | 0.00~0.68 | 0.18 | 0.13 |
| 5/17 | 0.20 | 0.01~0.73 | 0.20 | 0.14 |
