# Supplementary material for: Transmission dynamics and successful control measures of SARS-CoV-2 in the mega-size city of Guangzhou, China
Source: Medicine (Baltimore). 2021 Dec 3;100(48):e27846. doi: 10.1097/MD.0000000000027846 (PMC9191374; doi:10.1097/MD.0000000000027846)
Supplement: Supplemental Digital Content [file medi-100-e27846-s010.docx]

Supplementary Table 7. **Expected Number of Symptomatic SARS-CoV-2 Infections That Would Be Undetected During Active Monitoring,**

**Given Varying Monitoring Durations and Risks for Symptomatic Infection After Exposure***

| **Monitoring**  **Duration** | **Mean Estimated Number of Undetected Symptomatic Infections per 10 000 Monitored Persons (99th Percentile)** | | | |
| --- | --- | --- | --- | --- |
|  | **Low Risk**  **（1/10000）** | **Medium Risk**  **（1/1000）** | **High Risk**  **（1/100）** | **Infected**  **（1/1）** |
| 7 d | 0.4(0.5) | 3.9(4.8) | 38.7(47.7) | 3865.7(4769.3) |
| 14 d | 0.1(0.1) | 0.5(1.1) | 5.3(10.9) | 528.4(1092.1) |
| 21 d | 0.0(0.0) | 0.0(0.2) | 0.4(1.7) | 41.8(172.5) |
| 28 d | 0.0(0.0) | 0.0(0.0) | 0.0(0.2) | 2.7(23.0) |
| 35 d | 0.0(0.0) | 0.0(0.0) | 0.0(0.0) | 0.2(2.5) |
| 42 d | 0.0(0.0) | 0.0(0.0) | 0.0(0.0) | 0.0(0.2) |

SARS-CoV-2 = severe acute respiratory syndrome coronavirus 2.

* Estimates were generated from a probabilistic model using the incubation period estimates from the log-normal model.
